# *KRAS* p.G12C Mutation in Metastatic Colorectal Cancer: Prognostic Implications and Advancements in Targeted Therapies

**DOI:** 10.3390/cancers15143579

**Published:** 2023-07-12

**Authors:** Alessandro Ottaiano, Francesco Sabbatino, Francesco Perri, Marco Cascella, Roberto Sirica, Renato Patrone, Maurizio Capuozzo, Giovanni Savarese, Monica Ianniello, Nadia Petrillo, Luisa Circelli, Vincenza Granata, Massimiliano Berretta, Mariachiara Santorsola, Guglielmo Nasti

**Affiliations:** 1 Istituto Nazionale Tumori di Napoli, IRCCS “G. Pascale”, Via M. Semmola, 80131 Naples, Italy; f.perri@istitutotumori.na.it (F.P.); m.cascella@istitutotumori.na.it (M.C.); r.patrone@istitutotumori.na.it (R.P.); v.granata@istitutotumori.na.it (V.G.); mariachiara.santorsola@istitutotumori.na.it (M.S.); g.nasti@istitutotumori.na.it (G.N.); 2 Oncology Unit, Department of Medicine, Surgery and Dentistry, University of Salerno, Baronissi, 84081 Salerno, Italy; fsabbatino@unisa.it; 3 AMES, Centro Polidiagnostico Strumentale srl, Via Padre Carmine Fico 24, 80013 Casalnuovo Di Napoli, Italy; roberto.sirica@centroames.it (R.S.); giovanni.savarese@centroames.it (G.S.); monica.ianniello@centroames.it (M.I.); nadia.petrillo@centroames.it (N.P.); lulacir@libero.it (L.C.); 4 Coordinamento Farmaceutico, ASL-Naples-3, 80056 Ercolano, Italy; m.capuozzo@aslnapoli3sud.it; 5 Department of Clinical and Experimental Medicine, University of Messina, 98122 Messina, Italy; mberretta@unime.it

**Keywords:** KRAS, genetics, prognosis, p.G12C mutation, metastatic colorectal cancer

## Abstract

**Simple Summary:**

In this review, we aim to clarify the prognostic significance of the *KRAS* p.G12C mutation in metastatic colorectal cancer, emphasizing its potential as a promising therapeutic target. Moreover, our objective is to increase readers’ awareness of the factors that can influence diverse clinical, prognostic and therapeutic implications associated with distinct mutations within the same protein.

**Abstract:**

*KRAS* is frequently mutated in tumors. It is mutated in approximately 30% of all cancer cases and in nearly 50% of cases of metastatic colorectal cancer (CRC), which is the third leading cause of cancer-related deaths worldwide. Recent advancements in understanding CRC biology and genetics have highlighted the significance of *KRAS* mutations in the progression of CRC. The *KRAS* gene encodes a small GTPase (Guanosine TriPhosphatases) that plays a key role in signaling pathways associated with important proteins involved in amplifying growth factor and receptor signals. Mutations in *KRAS* are frequently observed in codons 12 and 13, and these mutations have oncogenic properties. Abnormal activation of KRAS proteins strongly stimulates signals associated with various cancer-related processes in CRC, including cell proliferation, migration and neoangiogenesis. In this review, we explore the distinct prognostic implications of *KRAS* mutations. Specifically, the *KRAS* p.G12C mutation is associated with a worse prognosis in metastatic CRC. The correlation between structure, conformation and mutations is visually presented to emphasize how alterations in individual amino acids at the same position in a single protein can unexpectedly exhibit complex involvement in cancer. Last, *KRAS* p.G12C is discussed as an emerging and promising therapeutic target in metastatic CRC, providing a concise overview of available clinical data regarding the use of new inhibitors.

## 1. Introduction

**Figure 1:**
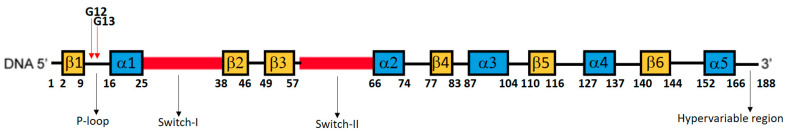
Schematic representation of amino acid positions, secondary structural elements, and functional elements of KRAS protein. The P-loop region (9–16), specifically positions 12 and 13, is the most common hotspot for mutations in *KRAS* gene, and it is essential for binding and hydrolyzing GTP. Switch 1 controls the conformational changes required for KRAS interaction with downstream effectors. Switch 2 plays a role in regulators and effectors binding. The hypervariable region is involved in membrane anchoring and localization of KRAS to specific cellular compartments. The GTP binding pocket is a complex structure formed through the interaction of amino acids from different regions (10–18, 57–63, 116–119, 143–146).

*KRAS* is a frequently mutated gene in tumors [1]. It is mutated in around 30% of all cancer cases and in nearly 50% of metastatic colorectal cancer (mCRC) cases. Colorectal cancer (CRC) is the third leading cause of cancer-related death worldwide [2]. Approximately half of patients present with or develop metastatic disease, primarily affecting the liver, lungs, lymph nodes and peritoneum. Unfortunately, despite recent advancements in mCRC treatment, patient survival rarely exceeds 30 months [3,4]. The increasing understanding of CRC biology and genetics has emphasized the significance of *KRAS*. The *KRAS* gene and its counterparts, K-RAS (Kirsten RAt Sarcoma viral oncogene homolog) and N-RAS (Neuroblastoma RAS viral oncogene homolog), encode small GTPases (Guanosine TriPhosphatases) that drive the signaling of EGFR (Epidermal Growth Factor Receptor) [5]. The *KRAS* gene encodes for a protein comprising 188 amino acid residues and has a mass of 21.6 kD. Figure 1 depicts a schematic representation of the KRAS protein and its functional components. It is beyond the scope of this review to provide an exhaustive account of the biochemistry of KRAS (metabolism, synthesis regulation, post-translational modifications, cellular localization, etc.). However, KRAS serves as a critical molecular switch, responsible for recruiting and activating essential proteins involved in mediating the signal transduction of growth factors and receptors, such as c-Raf, MAPK and PI 3-kinase. Upon activation, KRAS facilitates the hydrolysis of GTP to GDP, leading to the subsequent inactivation of KRAS [6]. The abnormal activation of RAS proteins strongly stimulates the signals associated with various cancer-related processes in CRC, including proliferation, migration and neoangiogenesis [7]. Furthermore, CRC tumors with *KRAS* mutations exhibit resistance to anti-EGFR therapies (cetuximab or panitumumab) because the EGFR pathway remains constitutively activated and independent of ligands in these cases [5,6,7].

## 2. *KRAS* Mutations: Prognostic Divergences

**Figure 2:**
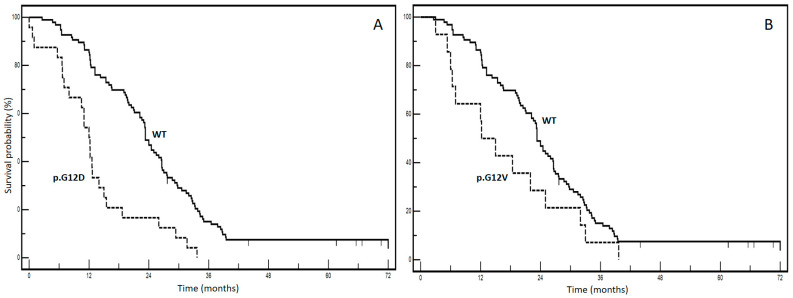

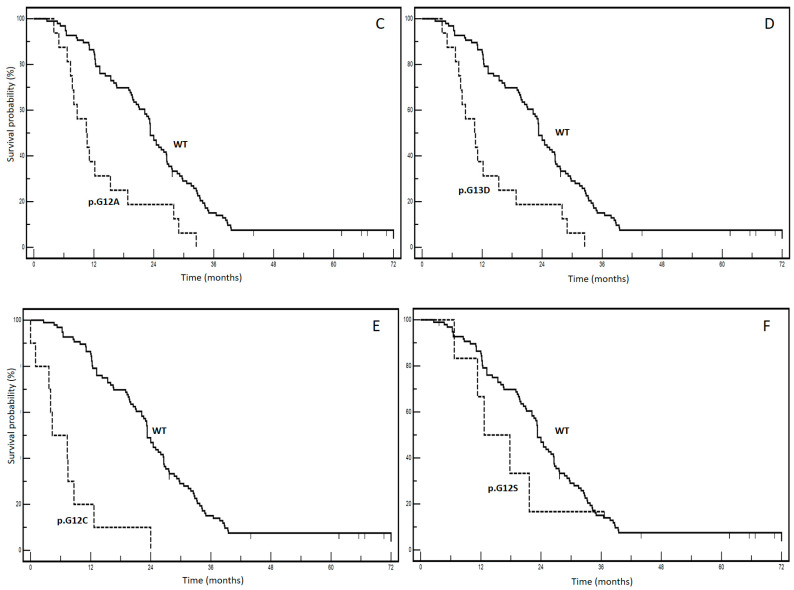
Kaplan–Meier survival curves according to different *KRAS* mutations ((**A**): WT vs. *KRAS* p.G12D; (**B**): WT vs. *KRAS* p.G12V; (**C**): WT vs. *KRAS* p. G13D; (**D**): WT vs. *KRAS* p.G13A; (**E**): WT vs. *KRAS* p.G12C; (**F**): WT vs. *KRAS* p.G13S). WT: wild-type KRAS patients.

From a prognostic standpoint, our previous report highlights the significant negative prognostic impact of pooled mutations involving *KRAS* p.G12C and p.G12S in patients with mCRC (STORIA study: Study of Ras mutations prognostic value in metastatic colorectal cancer) [8]. In an updated cohort of 188 patients from the STORIA study, who received homogeneous treatment at the SSD-Innovative Therapies for Abdominal Metastases, we conducted a prognostic analysis on individual mutational types (*KRAS* p.G12D, p.G12V, p.G13D, p.G12A, p.G12C, p.G12S). We chose to separate the *KRAS* variant categories to enable a more specific and useful examination of their prognostic power. Fourteen patients with rarer variants were excluded from the analysis to prevent potential prognostic interferences that could not be adequately explained due to limited and fragmented numbers (4 p.A146T, 2 p.A146V, 2 p.G13R, 2 p.K117N, 2 p.G13C, 1 p.G12_G13insG, 1 p.G12F). Table 1 presents the baseline clinicopathological characteristics of our case series. The overall survival for each mutation is illustrated in Figure 2 (panels A–F). Among these, panel E demonstrates the most significant divergence in survival curves, indicating a negative impact on prognosis for the *KRAS* p.G12C variant. The results of the univariate and multivariate analyses are summarized in Table 2.

No significant associations were found between *KRAS* mutational status and clinicopathological variables, as indicated in Table 1. Interestingly, in the multivariate analysis, adjusted for age, gender and metastatic involvement, p.G12V, p.G13D, p.G12A and p.G12S lost prognostic effect, whereas it was maintained by the p.G12D and p.G12C variants. The p.G12C variant had the worst prognostic profile with an impressive HR of 13.6 (95% CI: 3.9–17.16) and a median overall survival of 4.3 months compared to 23.3 months for the wt form (*p* < 0.0001). These findings provide compelling real-world evidence highlighting the clinical significance of the *KRAS* p.G12C variant in mCRC. They contribute to increasing awareness regarding how not all KRAS mutations have the same impact, highlighting the complexity of mCRC biology.

In recent studies, the prognostic significance of the *KRAS* p.G12C mutation in patients with mCRC has been investigated (Table 3). A search was performed in PubMed (last accessed on 10 April 2023) using the following search strings: (colon [title] OR colorectal [title]) AND (metasta*) AND (KRAS) AND (prognos*). The search was limited to the past ten years, and reviews were excluded. Additionally, the reference sections of the selected original papers were analyzed to ensure the inclusion of any additional relevant articles. Furthermore, our focus was specifically on studies that investigated the prognostic role of *KRAS* p.G12C and that reported the median survival of patients in months.

Various methodologies to assess *KRAS* mutations have been utilized, ranging from single-codon real-time PCR-based assays to Next-Generation Sequencing (NGS) wide panel assays. PCR-based kits and pyrosequencing-based assays offer high sensitivity and specificity in identifying *KRAS* mutations. They are relatively cost-effective and straightforward to perform. However, PCR-based kits can only detect pre-defined mutations and are unable to identify novel or rare mutations outside the targeted regions. Sanger sequencing is a well-established technique used for mutation detection, capable of identifying both single-nucleotide variants and small insertions or deletions. Nevertheless, it is time-consuming, expensive and, like the aforementioned techniques, not suitable for a high-throughput analysis. Next-Generation Sequencing (NGS) technologies have revolutionized genetic research, allowing for the simultaneous sequencing of numerous genes and enabling a comprehensive assessment of *KRAS* mutations and other genomic alterations. NGS offers high sensitivity, specificity and the ability to detect novel or rare mutations of the *KRAS* gene. However, the challenges associated with NGS include the cost, the complexity of the data analysis and the need for sophisticated bioinformatics tools. Interestingly, both the selected techniques and the heterogeneity in clinical series can contribute to significant variability in the frequency of *KRAS* p.G12C in the selected articles. These studies have unveiled the distinct prognostic implications of different *KRAS* mutations, contributing to the overall heterogeneity of tumors. Furthermore, to gain a better understanding of these findings, we can classify the studies into two groups. The first group compares prognostic outcomes among different mutations, and the second group compares mutated forms, whether singular or combined, with the non-mutated form. Notably, in our updated analysis of the STORIA study, we observed strong clinico-prognostic heterogeneity among *KRAS* mutations. Consequently, we found it valuable to compare the prognosis associated with each mutation to the non-mutated form, which we refer to as the “wt” (wild type). This comparison serves as a reference point for normalization, allowing for a more robust assessment of the prognostic significance of individual mutations.

Interestingly, in our study, we observed a median overall survival of 4.3 months for patients with *KRAS* p.G12C mutations. These findings are worse than those reported in a study with the poorest survival outcome for *KRAS* p.G12C mutated patients (15.2 months) [14]. Clinical factors related to the inherent heterogeneity of patient populations may account for this difference in prognoses. However, it is important to emphasize and discuss primarily the exclusion, in our study (STORIA trial), of oligometastatic patients (defined as those with 1–3 lesions per organ and the involvement of a maximum of two organs), which consistently represent 10% of the metastatic colorectal cancer cases. Furthermore, if we observe the median survival of *KRAS* wt patients in this study, it is evident that their survival is significantly higher (60.0 months) than that of ours (23.3 months), suggesting that this could be a contributing factor. Thus, in our study, the exclusion of oligometastatic patients and the presence of a higher proportion of poly-metastatic high tumor burden disease could have influenced the observed survival outcomes for *KRAS* p.G12C patients. It is crucial to consider these factors when interpreting and comparing our findings with previous studies.

However, studies examining the prognostic effect of *KRAS* p.G12C mutations, compared to non-p.G12C mutations, have consistently reported a decrease in median survival of 2–7.7 months among patients with non-p.G12C mutations [9,10,11]. Furthermore, when comparing the p.G12C mutation to the wt form of *KRAS*, studies have consistently demonstrated a significant negative impact, with median survival differences of less than 10 months compared to *KRAS* wt [12,13,14]. Therefore, the negative prognostic role of *KRAS* p.G12C in metastatic CRC is reasonably well-established.

It is important to acknowledge that our study has certain limitations. First, the sample size was relatively small, especially for certain *KRAS* mutations that occurred in less than 10 patients. This limited sample size could have influenced the robustness of our findings. Furthermore, due to the limited number of patients with rare *KRAS* variants, we were unable to thoroughly investigate the role of these variants, which might have had an even stronger prognostic impact. This is an important aspect that should be considered in future studies aiming to explore the prognostic significance of less common KRAS mutations. Additionally, the search conducted for the narrative review was not comprehensive but rather focused on providing a narrative overview of relevant studies. As a result, there is a possibility that some studies related to the topic may have been missed.

Despite these limitations, our study contributes to the growing body of evidence indicating that the *KRAS* p.G12C mutation is a strong negative prognostic factor in metastatic colorectal cancer. The consistent decrease in median survival observed in studies comparing p.G12C mutations to non-p.G12C mutations, as well as the significant negative impact when compared to the *KRAS* wt, strongly supports the unfavorable prognostic role of *KRAS* p.G12C in this patient population. These findings underscore the need for further insights and targeted therapeutic approaches in this patient population.

## 3. KRAS Mutations: Linking Structure and Prognosis

The awareness of certain connections between structure and prognostic implications is important. *KRAS* p.G12C is a paradigm of how a single protein can have unexpectedly complex involvement with a final effect linked not only to its alteration but also to the interplay with other molecular partners.

**Figure 3:**
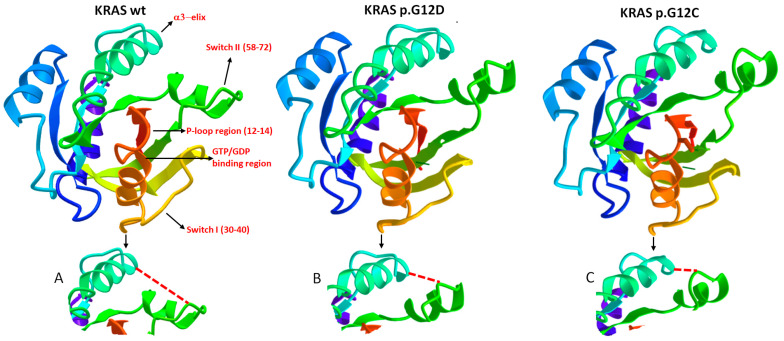
This figure illustrates three crystallographic structures of different KRAS variants: KRAS wild type, KRAS p.G12D and KRAS p.G12C. These variants differ by a single amino acid at position 12 in the P-loop region. It is important to note that, although a crystal structure represents an average of the protein structure over time, influenced by factors such as temperature, crystal-packing contacts and artifacts, it still provides a reliable approximation of the overall conformation of the protein regions. All three crystallographic structures were derived from the GDP-bound state of KRAS, and the analysis of single amino acid sequences and structural interactions between different regions was performed using the 3D navigation perspective on the online research tool of the NCBI (National Center for Biotechnology Information, https://www.ncbi.nlm.nih.gov/structure, accessed on 12 May 2023). In the figure, specific regions of interest are indicated, and ions and chemicals (such as glycerol, commonly used as a cryosolvent in cryocrystallography) are excluded to improve figure clarity. It is crucial to highlight that KRAS predominantly engages with its effector and regulatory proteins at the critical regions known as Switch I and Switch II. The GTP-binding site is located between the Switch I and P-loop regions. Notably, the depicted variants in the figure exhibit distinct allosteric conformations in the Switch II region (see Figures (**A**–**C**) below each representation of the respective KRAS variant). These structural differences contribute to the observed variations in oncogenic properties among the variants, implying distinct relationships with effectors and regulators, as well as differential GTPase activity.

Notably, KRAS hot spot mutations predominantly occur in codons 12 and 13. Mutations in codons 61 and 146, although oncogenic, account for less than 5% of total KRAS mutations [5,6]. The most significant consequence of alterations in codons 12 and 13 is the allosteric distortion of the GDP/GTP binding pocket, which leads to the abolition of or reduction in GTPase activity in KRAS following guanine nucleotide activating protein (GAP) binding. Additionally, this alteration causes the molecule to remain locked in an active, GTP-bound state. It is important to note that codons 12 and 13 in the KRAS wild type encode glycine residues. When other amino acids are substituted at these positions (e.g., aspartate and valine at codon 12, and aspartate at codon 13), bulky amino acid side chains protrude into the GDP/GTP binding pocket of KRAS, hindering the steric hindrance in GTP hydrolysis [15]. Nonetheless, the functional implications diverge significantly, particularly concerning the interaction interface with effector proteins. This disparity not only exists between the wt and mutant variants but also among different mutant forms. Intriguingly, among the various KRAS mutants, p.G12D, which is the most prevalent in cancer, exhibits the closest resemblance to the wt in terms of its dynamics [16]. These findings strongly suggest and validate that an alteration in KRAS dynamics follows an allosteric mechanism, and a mutation can induce diverse modifications in the protein, even in distal regions. Hence, structural variations in mutated forms of KRAS arise from allosteric effects on the protein, resulting in spatial distortions within regions involved in binding effectors and GAPs. These GAPs regulate GTP hydrolysis and facilitate the transition of KRAS into its inactive GDP-bound state [17]. As a result, these modifications in the three-dimensional structure of KRAS lead to differences in its ability to interact with effector and regulatory molecules. Figure 3 provides a comparative representation of the structures of the KRAS wt, KRAS p.G12D and p.G12C, demonstrating their distinct spatial conformations in specific regions.

Depending on the nature of the distortion, this conformational change can interfere variably with the binding of other molecular partners, especially those involved in KRAS deactivation. These considerations are crucial in understanding the diverse clinical and prognostic implications of *KRAS* mutations.

Studying *KRAS* mutations is further complicated by recent advancements in comprehending the molecular dynamics of individual mutations. Notably, *KRAS* p.G12C exhibits constitutive activation by remaining bound to GTP, leading to impaired GTP hydrolysis and insensitivity to p120RAS and neurofibromin (critical GAPs involved in GTPase activation). However, contrary to the prevailing belief that mutant KRAS p.G12C proteins are insensitive to GAPs, a paradox arises with the compound AMG510 (sotorasib), as it selectively recognizes only the inactive GDP-bound form of KRAS p.G12C. Interestingly, this paradox was resolved with the discovery of G protein signaling 3 (RGS3) as an unexpected GAP for KRAS p.G12C [18].

The activation of inactive KRAS-GDP is further complicated by the involvement of guanosine exchange factors (GEFs). GEF proteins facilitate the displacement of GDP from the nucleotide-binding site, ultimately resulting in the binding of GTP and the full activation of KRAS, including the specific variant KRAS p.G12C. Studies have demonstrated that the activation of KRAS p.G12C depends on receptor tyrosine kinase-mediated stimulation of the RAS guanine nucleotide exchange factor (RASGEF), SOS1 (son of seven-less homolog 1), which promotes the exchange of GDP for GTP. Therefore, inhibitors targeting SOS1 may hold therapeutic value against persistently activated KRAS p.G12C, in combination with direct inhibitors [19].

The biologic role of the *KRAS* p.G12C mutation varies among different types of cancers. This variability can be attributed to distinct tissue expression patterns and the involvement of regulatory proteins, such as SOS1, which may contribute to the differential response observed in KRAS p.G12C inhibition. An illustrative example of this variability is the response to sotorasib in metastatic non-small cell lung cancer, where an objective response is achieved in approximately 30% of cases, compared to approximately 7% of cases in colorectal cancer [20,21]. Additionally, in lung cancer, the *KRAS* p.G12C mutation is associated with high Tumor Mutational Burden (TMB) and high PD-L1 expression, suggesting potential increased sensitivity to immunotherapy [22].

Although the specific biological mechanisms underlying the differential effects of *KRAS* mutations are not yet fully understood, it has been demonstrated that the germline biallelic inactivation of the *MUTYH* (MutY Homolog) gene is associated with the occurrence of colorectal cancer bearing *KRAS* p.G12C and *PIK3CA* p.Q546K mutations [23]. MUTYH is an enzyme involved in the repair of DNA errors caused by guanine oxidation resulting from cellular oxidative stress. It functions as an adenine DNA glycosylase, specifically targeting and removing misincorporated adenine within 7,8-dihydro-8-oxoguanine (8-oxoG) pairs. This enzymatic action leads to G:C to T:A transversions. MUTYH works in collaboration with OGG1 (8-Oxoguanine DNA Glycosylase), which is responsible for eliminating 8-oxodG. Mutations in the *MUTYH* gene are associated with “MUTYH-associated polyposis syndrome” (MAP), an autosomal recessive disorder characterized by the development of multiple colorectal adenomas or polyps. Individuals with MAP have a significantly increased lifetime risk of developing colorectal cancer [24].

Understanding the mutational mechanisms of *KRAS* and delving into the role of different *KRAS* mutations in various tumor types present an intriguing challenge that can enhance our approach to agnostic therapies in oncology, with the inhibition of *KRAS* p.G12C serving as an example.

## 4. Resurgence in KRAS Targeting: Overcoming Challenges

**Figure 4:**
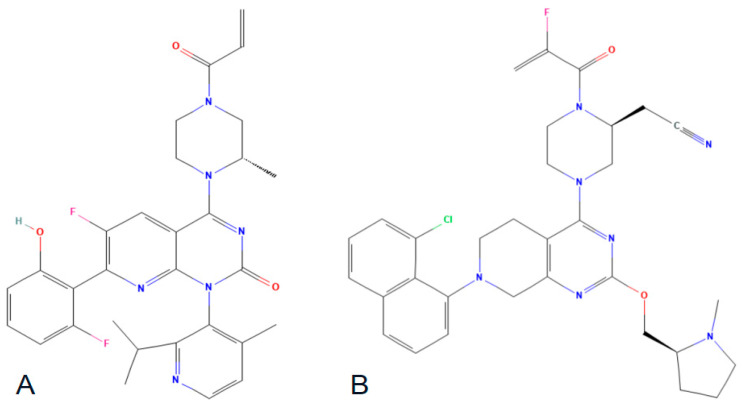
Chemical structures of sorotasib (**A**) and adagrasib (**B**).

Although KRAS has been identified as an excellent drug target for many cancers, the direct inhibition of oncogenic KRAS has proven to be challenging due to the absence of druggable pockets on its surface. Additionally, the development of direct KRAS inhibitors has faced difficulties due to the exceptionally high affinity of GTP and GDP to KRAS (with intracellular concentrations of these metabolites being much higher). However, in recent years, research on KRAS has experienced a resurgence, driven by the growing belief that KRAS could be targeted using low-molecular-weight organic molecules with a very high affinity for the protein. This belief was ignited by the discovery of two pockets on the surface of KRAS, particularly the Switch II pocket (SII-pocket) located above the Switch II loop in GDP-KRAS p.G12C, positioned between the α3-helix and Switch II loop [25,26,27]. These findings have revolutionized the pharmacological approach to targeting KRAS. Subsequently, a major breakthrough in KRAS inhibition for mCRC was achieved with the identification of covalent inhibitors specifically designed to target the p.G12C mutation in KRAS, namely sotorasib and adagrasib (Figure 4).

These compounds facilitate a covalent interaction between their electrophilic acryloyl components and the nucleophilic thiol group of the cysteine (Cys) residue at position 12. The KRAS p.G12C inhibitors have demonstrated encouraging outcomes in recent clinical trials; however, it is important to note, as previously discussed, that they specifically target the inactive form of KRAS.

## 5. Sotorasib and Adagrasib: An Overview of Clinical Data

The two drugs underwent clinical trials with promising results. In a phase I study, sotorasib was administered to 42 heavily pretreated and refractory patients with mCRC. The planned dose levels for the escalation cohorts were 180, 360, 720 and 960 mg once daily. The study achieved a response rate of 7.1% and a disease control rate of 73.8%. No dose-limiting toxic effects were observed, and no treatment-related adverse events resulted in death. The most common events were diarrhea (in 38 patients [29.5%]), fatigue (in 30 [23.3%]) and nausea (in 27 [20.9%]). The recommended dose for phase II studies was determined to be 960 mg per day [21]. In a phase II study involving 62 heavily pretreated and chemorefractory patients with the same disease, a response rate of 9.7% and a disease control rate of 82.3% were observed. The medication was given orally, with a daily dose of 960 mg, until there was evidence of disease progression, the occurrence of intolerable side effects, withdrawal of consent or mortality. Treatment-associated adverse events of grade 3 were observed in six patients (10%), predominantly presenting as diarrhea. There were no reported incidents of fatal outcomes [28]. In an interesting first-in-human phase I study of adagrasib, one of two heavily pretreated patients with KRAS p.G12C-mutant CRC achieved a partial response. The most common treatment-related adverse events of any grade were nausea (80.0%), diarrhea (70.0%), vomiting (50.0%) and fatigue (45.0%). Fatigue was the most common grade 3–4 treatment-related adverse event, occurring in 15.0% of patients. The recommended phase II dose based on safety, tolerability and observed pharmacokinetics properties was determined to be 600 mg twice a day [29]. In a non-randomized phase II clinical trial, heavily pretreated individuals diagnosed with mCRC harboring the KRAS p.G12C mutation were enrolled. The patients received either adagrasib monotherapy (600 mg orally twice daily) or a combination treatment comprising adagrasib (at the same dosage) and intravenous cetuximab once a week (at standard doses). Among the 44 patients who received adagrasib monotherapy, a response rate of 19% was observed. In the group receiving combination therapy (consisting of 32 patients), the response rate increased to 46%. Notably, treatment-related adverse events of grade 3 or 4 occurred in 34% of the monotherapy group and 16% of the combination therapy group. No grade 5 adverse events were reported.

Therefore, adagrasib and sotorasib exhibited comparable safety profiles and encouraging efficacy in extensively treated individuals diagnosed with mCRC carrying the G12C mutation in the KRAS gene. Combination with anti-EGFR therapy appears to improve clinical outcomes [30].

Sotorasib received orphan drug designation from the US FDA in June 2019 for *KRAS* p.G12C-positive non-small cell lung cancer (NSCLC) and colorectal cancer. However, as of the time of writing this manuscript, both sotorasib and adagrasib have only received FDA approval for NSCLC. Currently, phase I/II clinical trials are underway in multiple countries to evaluate the efficacy of sotorasib and adagrasib in *KRAS* p.G12C-mutated mCRC. These trials involve combinations with various treatment modalities, such as immunotherapy (NCT03785249), SHP-2 inhibitors (NCT04330664), ULK 1/2 kinases inhibitors (NCT04892017), anti-EGFR therapies (NCT05722327, NCT04793958, NCT05198934) and SOS-2 inhibitors (NCT05578092).

## 6. Pragmatic Considerations on the Clinical Use of KRAS Mutations

In clinical practice, oncologists receive a molecular report indicating a specific *KRAS* mutation. The primary consequence is the exclusion of anti-EGFR-based treatments, as widely recommended by guidelines [31]. Nevertheless, it is crucial to acknowledge and remain aware that not all *KRAS* mutations carry the same prognostic implications. For example, the presence of the p.G12C mutation may indicate a poorer prognosis, whereas the p.G12D mutation may suggest an intermediate outcome between the wt form and the p.G12C mutation. On the other hand, the p.G12V variant might exhibit a prognostic behavior that is not significantly different from that in patients with wt *KRAS*. Moreover, for several other mutations (p.A146T, 2 p.A146V, 2 p.G13R, 2 p.K117N, 2 p.G13C, 1 p.G12_G13insG, 1 p.G12F), due to their rarity, we have limited knowledge regarding their predictive and prognostic consequences. In such cases, we can only reasonably adopt a cautious approach and encourage enrollment in clinical studies or stimulate multi-institutional research to gather an adequate amount of data. This highlights the complexity of prognostic evaluations in mCRC, which already involve various clinical factors such as age, tumor burden, response to first-line chemotherapy, CEA levels, the site of the tumor, lymph node involvement, grading, histology and others. In the future, clinical oncologists will increasingly utilize advanced tools, such as artificial-intelligence-based systems, to enhance prognostic and therapeutic assessments. These tools will include the integration of molecular signatures into comprehensive and final prognostic evaluations. AI can provide enhanced decision support, leveraging the growing availability of data and technological advancements.

Further studies are necessary to deepen our understanding of the prognostic and therapeutic implications associated with different *KRAS* mutations in mCRC patients. These investigations will contribute to refining our knowledge and optimizing treatment strategies for this complex disease.

## 7. Conclusions

The *KRAS* p.G12C mutation holds significant clinical importance in patients with metastatic colorectal cancer. Investigating and extensively exploiting it as a therapeutic target is one of the priorities in oncology for the near future.

## Figures and Tables

**Table 1:** Clinical and pathological characteristics of patients.

Characteristics	WT	Mutated	*p*	Mutation						*p*
				p.G12D	p.G12V	p.G13D	p.G12A	p.G12C	p.G12S	
Age										
<65 y	50	40		9	9	11	3	5	3	
≥65 y	46	38	0.91	15	5	5	4	5	4	0.40
Gender										
M	49	35		10	8	5	5	4	3	
F	47	43	0.65	14	6	11	2	6	4	0.51
Grading										
G1/G2	20	17		4	3	5	2	1	2	
G3	76	61	0.87	20	11	11	5	9	5	0.79
Side of primary tumor										
Left	45	32		9	8	8	1	3	3	
Right	51	46	0.44	15	6	8	6	7	4	0.44
pT										
pT1/pT2	13	11		4	1	3	2	1	0	
pT3	48	45		12	11	8	2	7	5	
pT4	35	22	0.50	8	2	5	3	2	2	0.63
pN										
Not Involved	21	23		8	4	6	3	0	2	
Involved	75	55	0.25	16	10	10	4	10	5	0.36
Metastatic involvement										
One site	23	25		9	6	4	3	1	2	
More than one	73	53	0.23	15	8	12	4	9	5	0.53
No. of chemotherapy lines										
Two	10	16		3	3	3	2	2	3	
More than two	86	62	0.06	21	11	13	5	8	4	0.63

**Table 2:** Uni- and multivariate analysis of *KRAS* mutations’ prognostic effect.

Characteristics	Risk Factor (vs. Comparator)	mOS (Months)	No. of Events/Patients	*p* at Univariate	HR	95% CI	*p* at Multivariate
Age	<65 y (vs. ≥65 y)	15.5 (vs. 19.5)	87/90 (vs. 77/84)	0.1310	0.79	0.59–1.07	0.0965
Gender	male (vs. female)	19.2 (vs. 15.4)	81/84 (vs. 83/90)	0.7870	1.04	0.77–1.39	0.1181
Metastatic involvement	>1 site (vs. 1 site)	13.2 (vs. 23.6)	132/138 (vs. 32/36)	0.0005	1.77	1.28–2.45	<0.0001
KRAS mutations	p.G12D (vs. WT)	12.0 (vs. 23.3)	24/24 (vs. 89/96)	<0.0001	4.88	2.52–9.46	<0.0001
	p.G12V (vs. WT)	12.2 (vs. 23.3)	14/14 (vs. 89/96)	0.0508	2.01	0.99–4.08	0.0745
	p.G13D (vs. WT)	10.5 (vs. 23.3)	16/16 (vs. 89/96)	0.0253	5.49	2.41–12.4	0.0927
	p.G12A (vs. WT)	12.5 (vs. 23.3)	6/7 (vs. 89/96)	0.4127	1.50	0.56–4.00	0.2656
	p.G12C (vs. WT)	4.3 (vs. 23.3)	10/10 vs. (89/96)	<0.0001	13.6	3.9–17.16	<0.0001
	p.G12S (vs. WT)	12.6 (vs. 23.3)	5/7 vs. (89/96)	0.3541	1.68	0.56–5.05	0.1748

CI: Confidence Interval; HR: Hazard Ratio; mOS: Median Overall Survival.

**Table 3:** Survey of studies reporting on the prognostic role of *KRAS* p.G12C variant in mCRC patients.

Author, Year	Type of Study	Method	No. of Patients	% of p.G12C Variant	Comparison	mOSs	Co-Variates	*p* *
Schirripa M. et al., 2020 [9]	Retrospective	Sanger sequencing.	839	17.0	p.G12C vs. non-p.G12C	29.0 vs. 36.7	Gender, ECOG PS, primary tumor surgery, pT, pN, time of first metastasis, grading, number of metastatic sites.	0.004
Chida K. et al., 2021 [10]	Retrospective	Exon 2 through PCR-based kits.	1632	2.8	p.G12C vs. non-p.G12C	21.2 vs. 27.3	Age, gender, ECOG PS, primary tumor site, surgery on the primary tumor, time of first metastasis, histology, white blood cell count, serum albumin level, LDH level, serum C-reactive protein level, metastatic tumor site, number of metastatic sites.	0.030
Fakih M. et al., 2022 [11]	Retrospective	NGS.	6477	3.7	p.G12C vs. non-p.G12C vs. wt	16.1 vs. 18.3 vs. 23.4	None **	None **
Modest D.P. et al., 2016 [12]	Retrospective	Exon 2-4 through PCR-based kits.	1239	2.2	p.G12C vs. wt	16.8 vs. 26.9	Treatment, ECOG PS, gender, adjuvant chemotherapy, liver-limited disease and number of involved organs.	0.001
Jones R.P. et al., 2017 [13]	Retrospective	Pyrosequencing-based assay of codons 12, 13 and 61.	392	3.8	p.G12C vs. wt	24.9 vs. 35.1	None **	None **
Wiesweg M. et al., 2019 [14]	Retrospective	Exon 2, if KRAS wt proceeded to exon 3 through PCR-based kits.	347	11.9	p.G12C (included in an unfavorable cohort) vs. wt	15.2 vs. 60.0	Age, gender, stage, RAS prognostic cluster or RAS wild type, RAS DNA substitution category, grading, primary tumor location.	0.087

* The reported *p* refers to multivariable analysis results. ** The studies are descriptive. Co-variates of multivariable models are also reported. ECOG PS: Eastern Cooperative Oncology Group Performance Status; mOS: Median Overall Survival; NGS: Next-Generation Sequencing; PCR: Polymerase Chain Reaction.

## Data Availability

The methodology for conducting patient follow-up on vital status is reported at https://zenodo.org/deposit/8027561, last accessed on 10 July 2023.
